# Bis(μ-2-phenyl­quinoline-4-carboxyl­ato)-κ^3^ *O*,*O*′:*O*;κ^3^ *O*:*O*,*O*′-bis­[(2,2′-bipyridine-κ^2^ *N*,*N*′)(2-phenyl­quinoline-4-carboxyl­ato-κ^2^ *O*,*O*′)cadmium(II)]

**DOI:** 10.1107/S1600536810049640

**Published:** 2010-12-04

**Authors:** Mei-Qin Zha, Xing Li, Yue Bing

**Affiliations:** a Faculty of Materials Science and Chemical Engineering, Ningbo University, Ningbo, Zhejiang 315211, People’s Republic of China

## Abstract

The neutral binuclear title complex, [Cd_2_(C_16_H_10_NO_2_)_4_(C_10_H_8_N_2_)_2_], is centrosymmetric, with the inversion center generating the central (μ-O)_2_Cd_2_ bridge. The Cd^II^ ion is in a strongly distorted CdN_2_O_5_ penta­gonal-bipyramidal geometry, defined by two N atoms from one 2,2′-bipyridine ligand and five O atoms from three 2-phenyl­quinoline-4-carboxyl­ate ligands, one monodentate, two bidentate. Weak inter­molecular π–π inter­actions [centroid–centroid distance = 3.712 (3) Å] help to establish the packing of the structure.

## Related literature

For complexes including 2-phenyl­quinoline-4-carboxyl­ate as a ligand, see: Che *et al.* (2005 ▶); Qin *et al.* (1999 ▶, 2002 ▶); Shen *et al.* (2007 ▶); Zhang *et al.* (2009 ▶).
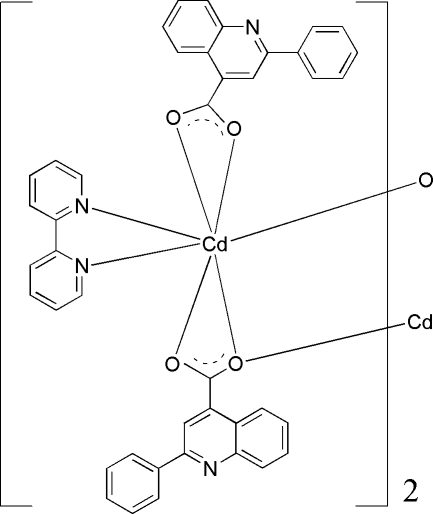


## Experimental

### 

#### Crystal data

[Cd_2_(C_16_H_10_NO_2_)_4_(C_10_H_8_N_2_)_2_]
*M* *_r_* = 1530.17
Triclinic, 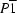

*a* = 10.000 (2) Å
*b* = 13.047 (3) Å
*c* = 13.157 (3) Å
α = 91.513 (3)°
β = 97.130 (3)°
γ = 95.185 (3)°
*V* = 1695.1 (6) Å^3^
*Z* = 1
Mo *K*α radiation
μ = 0.70 mm^−1^
*T* = 298 K
0.32 × 0.21 × 0.16 mm

#### Data collection

Bruker SMART APEX CCD area-detector diffractometer
Absorption correction: multi-scan (*SADABS*; Bruker, 2001 ▶) *T*_min_ = 0.839, *T*_max_ = 0.895
14431 measured reflections
7492 independent reflections
5547 reflections with *I* > 2σ(*I*)
*R*_int_ = 0.032

#### Refinement

*R*[*F* ^2^ > 2σ(*F* ^2^)] = 0.045
*wR*(*F* ^2^) = 0.134
*S* = 1.02
7492 reflections
460 parameters
H-atom parameters constrained
Δρ_max_ = 1.07 e Å^−3^
Δρ_min_ = −1.36 e Å^−3^

### 

Data collection: *SMART* (Bruker, 2001 ▶); cell refinement: *SAINT* (Bruker, 2001 ▶); data reduction: *SAINT*; program(s) used to solve structure: *SHELXS97* (Sheldrick, 2008 ▶); program(s) used to refine structure: *SHELXL97* (Sheldrick, 2008 ▶); molecular graphics: *SHELXTL-Plus* (Sheldrick, 2008 ▶) and *DIAMOND* (Brandenburg, 2006 ▶); software used to prepare material for publication: *SHELXL97*.

## Supplementary Material

Crystal structure: contains datablocks I, global. DOI: 10.1107/S1600536810049640/bh2323sup1.cif

Structure factors: contains datablocks I. DOI: 10.1107/S1600536810049640/bh2323Isup2.hkl

Additional supplementary materials:  crystallographic information; 3D view; checkCIF report

## Figures and Tables

**Table 1:** Selected geometric parameters (Å, °)

Cd1—N4	2.328 (3)
Cd1—O2^i^	2.336 (3)
Cd1—N3	2.338 (3)
Cd1—O2	2.338 (3)
Cd1—O3	2.349 (3)
Cd1—O4	2.465 (3)
Cd1—O1	2.618 (3)

Symmetry code: (i) 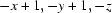.

**Fig. 1.**
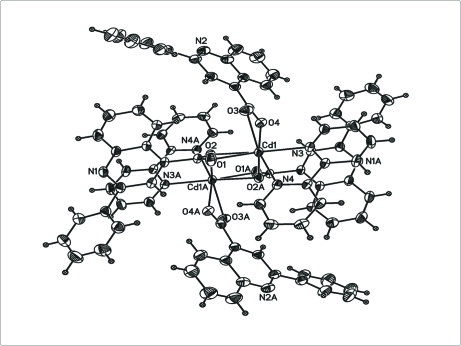
A view of the title compound with displacement ellipsoids drawn at the 30% probability level [symmetry code: (A) -x + 1, -y + 1, -z].

**Fig. 2.**
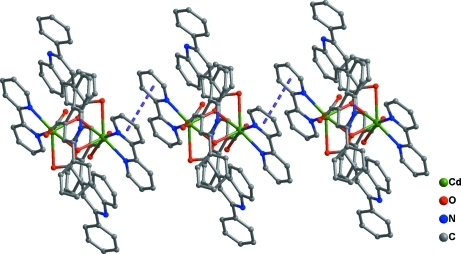
One-dimensional structure of the title complex..

## supplementary crystallographic information

### Comment

2-Phenylquinoline-4-carboxylic acid (Hphqc) is a potential multi-functional ligand containing carboxylate group and nitrogen coordination donors, and possesses rich coordination and structural chemistry. Few coordination compounds including 2-phenylquinoline-4-carboxylates have been assembled over twenty years (Che *et al.*, 2005, Qin *et al.*, 1999, 2002, Shen *et al.*, 2007, Zhang *et al.*, 2009). To the best of our knowledge, Cd(II) complexes with Hphqc have not been reported up to now. Herein, we report a new compound constructed from Cd(II) ions and (phqc)^-^ ligands.

The title compound reveals to be a centrosymmetric binuclear Cd(II) complex, as shown in Fig. 1. The Cd atom is seven-coordinated in a pentagonal bipyramidal geometry. The Cd(II) center is coordinated to two N donors from one 2,2'-bipyridine ligand and five O atoms from three (phqc)^-^ ligands. The Cd—O bond lengths are ranging from 2.336 (3) to 2.618 (3) Å, and Cd—N distance are 2.328 (3) and 2.338 (3) Å.

In complex 1, the centroid-to-centroid separation of the nearest two benzene rings of the 2,2'-bipyridine (N3, C33, C34, C35, C36, C37) and (N4, C38, C39, C40, C41, C42) is 3.712 Å, which implies intermolecular π–π stacking interaction. The dinuclear Cd(II) units are linked through these interactions to generate a one-dimensional chain (Fig. 2).

### Experimental

2-phenylquinoline-4-carboxylic acid (0.0240 g, 0.10 mmol), 3CdSO_4_.8H_2_O (0.0774 g, 0.10 mmol), 2,2'-bipyridine (0.0156 g, 0.10 mmol) and KOH (0.0056 g, 0.10 mmol) in H_2_O (10 ml) were placed in a 25 ml stainless reactor fitted with a Teflon liner and heated to 373 K (100 °C) for two days, and then cooled to room temperature. Colorless block-like crystals were obtained and dried in air. Yield: 76%, based on Cd.

### Refinement

H atoms attached to C atoms were placed in calculated positions and treated using a riding-model approximation, with C–H = 0.93 Å and *U*_iso_(H) = 1.2*U*_eq_(carrier C atom)].

### Crystal data

**Table:**

[Cd_2_(C_16_H_10_NO_2_)_4_(C_10_H_8_N_2_)_2_]	*Z* = 1
*M**_r_* = 1530.17	*F*(000) = 776
Triclinic, *P*1	*D*_x_ = 1.499 Mg m^−^^3^
Hall symbol: -P 1	Mo *K*α radiation, λ = 0.71073 Å
*a* = 10.000 (2) Å	Cell parameters from 14431 reflections
*b* = 13.047 (3) Å	θ = 1.6–27.5°
*c* = 13.157 (3) Å	µ = 0.70 mm^−^^1^
α = 91.513 (3)°	*T* = 298 K
β = 97.130 (3)°	Block, colourless
γ = 95.185 (3)°	0.32 × 0.21 × 0.16 mm
*V* = 1695.1 (6) Å^3^	

### Data collection

**Table:**

Bruker SMART APEX CCD area-detector diffractometer	7492 independent reflections
Radiation source: fine-focus sealed tube	5547 reflections with *I* > 2σ(*I*)
graphite	*R*_int_ = 0.032
φ and ω scans	θ_max_ = 27.5°, θ_min_ = 1.6°
Absorption correction: multi-scan (*SADABS*; Bruker, 2001)	*h* = −12→12
*T*_min_ = 0.839, *T*_max_ = 0.895	*k* = −16→16
14431 measured reflections	*l* = −17→16

### Refinement

**Table:**

Refinement on *F*^2^	Primary atom site location: structure-invariant direct methods
Least-squares matrix: full	Secondary atom site location: difference Fourier map
*R*[*F*^2^ > 2σ(*F*^2^)] = 0.045	Hydrogen site location: inferred from neighbouring sites
*wR*(*F*^2^) = 0.134	H-atom parameters constrained
*S* = 1.02	*w* = 1/[σ^2^(*F*_o_^2^) + (0.0731*P*)^2^ + 0.665*P*] where *P* = (*F*_o_^2^ + 2*F*_c_^2^)/3
7492 reflections	(Δ/σ)_max_ < 0.001
460 parameters	Δρ_max_ = 1.07 e Å^−^^3^
0 restraints	Δρ_min_ = −1.36 e Å^−^^3^
0 constraints	

### Fractional atomic coordinates and isotropic or equivalent isotropic displacement parameters (Å^2^)

**Table:**

	*x*	*y*	*z*	*U*_iso_*/*U*_eq_	
Cd1	0.33778 (3)	0.47866 (2)	0.05568 (2)	0.03810 (11)	
O1	0.3538 (3)	0.6793 (2)	0.0437 (3)	0.0587 (8)	
O2	0.5332 (3)	0.59229 (19)	0.0598 (2)	0.0443 (7)	
O3	0.3779 (3)	0.5140 (3)	0.2334 (2)	0.0564 (8)	
O4	0.4866 (3)	0.3875 (2)	0.1788 (2)	0.0549 (8)	
N1	0.7437 (4)	0.9514 (3)	0.0256 (3)	0.0504 (9)	
N2	0.7618 (4)	0.5106 (3)	0.5176 (3)	0.0562 (10)	
N3	0.1677 (3)	0.3442 (2)	0.0602 (3)	0.0403 (7)	
N4	0.1683 (3)	0.4902 (2)	−0.0807 (3)	0.0395 (7)	
C1	0.4769 (4)	0.6758 (3)	0.0501 (3)	0.0400 (9)	
C2	0.5700 (4)	0.7730 (3)	0.0457 (3)	0.0375 (8)	
C3	0.5460 (4)	0.8372 (3)	−0.0337 (3)	0.0414 (9)	
H3	0.4697	0.8230	−0.0818	0.050*	
C4	0.6858 (4)	0.7968 (3)	0.1193 (3)	0.0405 (9)	
C5	0.7220 (4)	0.7366 (3)	0.2047 (3)	0.0475 (10)	
H5	0.6710	0.6748	0.2129	0.057*	
C6	0.8309 (5)	0.7682 (4)	0.2750 (4)	0.0632 (13)	
H6	0.8529	0.7284	0.3312	0.076*	
C7	0.9095 (6)	0.8602 (5)	0.2629 (4)	0.0792 (17)	
H7	0.9831	0.8814	0.3116	0.095*	
C8	0.8799 (5)	0.9186 (4)	0.1813 (4)	0.0712 (15)	
H8	0.9342	0.9790	0.1741	0.085*	
C9	0.7676 (4)	0.8892 (3)	0.1063 (3)	0.0481 (10)	
C10	0.6371 (4)	0.9258 (3)	−0.0429 (3)	0.0443 (10)	
C11	0.6183 (5)	0.9933 (3)	−0.1331 (3)	0.0480 (10)	
C12	0.4954 (5)	0.9937 (4)	−0.1943 (4)	0.0604 (12)	
H12	0.4216	0.9498	−0.1811	0.073*	
C13	0.4821 (6)	1.0595 (4)	−0.2756 (4)	0.0757 (16)	
H13	0.3986	1.0598	−0.3155	0.091*	
C14	0.7255 (6)	1.0582 (4)	−0.1567 (4)	0.0626 (13)	
H14	0.8097	1.0582	−0.1177	0.075*	
C15	0.7097 (7)	1.1232 (4)	−0.2373 (4)	0.0804 (17)	
H15	0.7829	1.1677	−0.2508	0.097*	
C16	0.5885 (7)	1.1232 (4)	−0.2977 (5)	0.0800 (17)	
H16	0.5791	1.1661	−0.3530	0.096*	
C17	0.4680 (4)	0.4531 (4)	0.2459 (3)	0.0482 (10)	
C18	0.5663 (4)	0.4659 (3)	0.3445 (3)	0.0456 (10)	
C19	0.5886 (4)	0.5610 (3)	0.3926 (3)	0.0474 (10)	
H19	0.5359	0.6129	0.3694	0.057*	
C20	0.6428 (5)	0.3864 (4)	0.3859 (3)	0.0500 (10)	
C21	0.6304 (5)	0.2832 (4)	0.3474 (4)	0.0633 (13)	
H21	0.5714	0.2641	0.2882	0.076*	
C22	0.7046 (6)	0.2104 (4)	0.3963 (5)	0.0755 (16)	
H22	0.6952	0.1429	0.3701	0.091*	
C23	0.7935 (7)	0.2381 (5)	0.4849 (5)	0.0823 (17)	
H23	0.8425	0.1885	0.5178	0.099*	
C24	0.8095 (6)	0.3356 (5)	0.5233 (4)	0.0782 (16)	
H24	0.8692	0.3522	0.5826	0.094*	
C25	0.7377 (5)	0.4129 (4)	0.4756 (3)	0.0548 (11)	
C26	0.6904 (5)	0.5819 (4)	0.4768 (3)	0.0502 (10)	
C27	0.7241 (5)	0.6903 (4)	0.5209 (3)	0.0539 (11)	
C28	0.8561 (6)	0.7209 (5)	0.5635 (4)	0.0736 (15)	
H28	0.9209	0.6739	0.5683	0.088*	
C29	0.8908 (7)	0.8215 (5)	0.5986 (5)	0.0889 (19)	
H29	0.9800	0.8427	0.6248	0.107*	
C30	0.6305 (6)	0.7601 (4)	0.5161 (4)	0.0666 (14)	
H30	0.5425	0.7402	0.4861	0.080*	
C31	0.6634 (7)	0.8600 (5)	0.5549 (5)	0.0878 (18)	
H31	0.5972	0.9058	0.5535	0.105*	
C32	0.7940 (8)	0.8911 (5)	0.5953 (5)	0.097 (2)	
H32	0.8176	0.9586	0.6202	0.116*	
C33	0.1753 (4)	0.2716 (3)	0.1293 (3)	0.0498 (10)	
H33	0.2515	0.2747	0.1781	0.060*	
C34	0.0750 (5)	0.1920 (3)	0.1319 (4)	0.0598 (13)	
H34	0.0844	0.1414	0.1801	0.072*	
C35	−0.0381 (5)	0.1893 (3)	0.0622 (4)	0.0626 (13)	
H35	−0.1080	0.1373	0.0629	0.075*	
C36	−0.0483 (4)	0.2644 (3)	−0.0096 (4)	0.0526 (11)	
H36	−0.1253	0.2639	−0.0572	0.063*	
C37	0.0583 (4)	0.3412 (3)	−0.0099 (3)	0.0377 (8)	
C38	0.0570 (4)	0.4227 (3)	−0.0875 (3)	0.0359 (8)	
C39	−0.0508 (4)	0.4296 (3)	−0.1635 (3)	0.0485 (10)	
H39	−0.1288	0.3846	−0.1659	0.058*	
C40	−0.0408 (5)	0.5043 (4)	−0.2358 (3)	0.0555 (11)	
H40	−0.1122	0.5098	−0.2872	0.067*	
C41	0.0742 (5)	0.5695 (4)	−0.2312 (3)	0.0551 (11)	
H41	0.0834	0.6188	−0.2803	0.066*	
C42	0.1766 (4)	0.5613 (3)	−0.1527 (3)	0.0495 (10)	
H42	0.2546	0.6066	−0.1492	0.059*	

### Atomic displacement parameters (Å^2^)

**Table:**

	*U*^11^	*U*^22^	*U*^33^	*U*^12^	*U*^13^	*U*^23^
Cd1	0.03085 (17)	0.03765 (16)	0.04228 (18)	−0.00643 (11)	−0.00203 (11)	0.00059 (11)
O1	0.0335 (17)	0.0550 (18)	0.084 (2)	−0.0065 (13)	−0.0006 (16)	0.0042 (16)
O2	0.0529 (18)	0.0303 (13)	0.0496 (16)	−0.0019 (12)	0.0107 (14)	−0.0036 (11)
O3	0.0427 (18)	0.078 (2)	0.0460 (17)	0.0069 (16)	−0.0041 (14)	0.0008 (15)
O4	0.058 (2)	0.0571 (18)	0.0453 (17)	−0.0036 (15)	−0.0040 (15)	−0.0029 (15)
N1	0.052 (2)	0.0368 (17)	0.059 (2)	−0.0091 (15)	0.0023 (18)	0.0033 (16)
N2	0.051 (2)	0.072 (3)	0.043 (2)	0.010 (2)	−0.0053 (18)	0.0021 (19)
N3	0.0346 (18)	0.0385 (16)	0.0455 (19)	−0.0024 (13)	0.0012 (15)	−0.0019 (14)
N4	0.0334 (18)	0.0400 (16)	0.0432 (18)	0.0003 (13)	0.0003 (14)	−0.0008 (14)
C1	0.041 (2)	0.0329 (18)	0.043 (2)	−0.0083 (16)	0.0033 (18)	−0.0039 (16)
C2	0.035 (2)	0.0288 (17)	0.048 (2)	−0.0015 (15)	0.0059 (17)	−0.0053 (16)
C3	0.037 (2)	0.0348 (18)	0.050 (2)	0.0010 (16)	0.0003 (18)	−0.0062 (17)
C4	0.038 (2)	0.0336 (18)	0.048 (2)	−0.0030 (16)	0.0056 (18)	−0.0026 (16)
C5	0.050 (3)	0.043 (2)	0.047 (2)	−0.0047 (18)	0.003 (2)	0.0003 (18)
C6	0.065 (3)	0.063 (3)	0.057 (3)	−0.002 (2)	−0.009 (2)	0.011 (2)
C7	0.063 (4)	0.087 (4)	0.074 (4)	−0.024 (3)	−0.027 (3)	0.010 (3)
C8	0.061 (3)	0.065 (3)	0.076 (4)	−0.026 (3)	−0.014 (3)	0.009 (3)
C9	0.043 (2)	0.041 (2)	0.056 (3)	−0.0076 (18)	−0.003 (2)	0.0008 (19)
C10	0.047 (3)	0.0312 (18)	0.053 (2)	0.0023 (17)	0.004 (2)	−0.0011 (17)
C11	0.057 (3)	0.0338 (19)	0.053 (3)	0.0030 (18)	0.009 (2)	−0.0008 (18)
C12	0.065 (3)	0.050 (2)	0.067 (3)	0.010 (2)	0.005 (3)	0.007 (2)
C13	0.088 (4)	0.070 (3)	0.072 (4)	0.030 (3)	0.000 (3)	0.015 (3)
C14	0.069 (3)	0.055 (3)	0.061 (3)	−0.006 (2)	0.005 (3)	0.005 (2)
C15	0.108 (5)	0.062 (3)	0.071 (4)	−0.012 (3)	0.023 (4)	0.016 (3)
C16	0.110 (5)	0.062 (3)	0.072 (4)	0.013 (3)	0.018 (4)	0.024 (3)
C17	0.041 (2)	0.058 (3)	0.043 (2)	−0.010 (2)	0.0041 (19)	0.009 (2)
C18	0.039 (2)	0.059 (3)	0.037 (2)	−0.0002 (19)	0.0030 (18)	0.0051 (19)
C19	0.040 (2)	0.061 (3)	0.041 (2)	0.0045 (19)	0.0026 (19)	0.005 (2)
C20	0.045 (3)	0.060 (3)	0.044 (2)	0.001 (2)	0.007 (2)	0.005 (2)
C21	0.065 (3)	0.067 (3)	0.054 (3)	0.003 (3)	−0.005 (2)	−0.003 (2)
C22	0.087 (4)	0.062 (3)	0.076 (4)	0.016 (3)	0.000 (3)	0.002 (3)
C23	0.092 (5)	0.079 (4)	0.075 (4)	0.030 (3)	−0.009 (3)	0.008 (3)
C24	0.086 (4)	0.082 (4)	0.062 (3)	0.022 (3)	−0.017 (3)	0.001 (3)
C25	0.057 (3)	0.066 (3)	0.042 (2)	0.013 (2)	0.001 (2)	0.007 (2)
C26	0.045 (3)	0.066 (3)	0.038 (2)	0.002 (2)	0.0037 (19)	−0.003 (2)
C27	0.057 (3)	0.060 (3)	0.043 (2)	0.004 (2)	0.001 (2)	−0.001 (2)
C28	0.072 (4)	0.079 (4)	0.063 (3)	0.002 (3)	−0.012 (3)	−0.009 (3)
C29	0.081 (4)	0.091 (4)	0.085 (4)	−0.013 (4)	−0.007 (3)	−0.028 (4)
C30	0.065 (3)	0.071 (3)	0.063 (3)	0.003 (3)	0.008 (3)	−0.007 (3)
C31	0.093 (5)	0.075 (4)	0.095 (5)	0.012 (3)	0.007 (4)	−0.015 (3)
C32	0.118 (6)	0.076 (4)	0.090 (5)	−0.010 (4)	0.005 (4)	−0.026 (4)
C33	0.044 (3)	0.049 (2)	0.054 (3)	−0.0040 (19)	−0.001 (2)	0.004 (2)
C34	0.062 (3)	0.044 (2)	0.070 (3)	−0.010 (2)	0.004 (3)	0.014 (2)
C35	0.053 (3)	0.044 (2)	0.085 (4)	−0.017 (2)	0.002 (3)	0.009 (2)
C36	0.036 (2)	0.043 (2)	0.073 (3)	−0.0075 (18)	−0.007 (2)	−0.004 (2)
C37	0.031 (2)	0.0335 (18)	0.047 (2)	0.0013 (15)	0.0036 (17)	−0.0087 (16)
C38	0.0278 (19)	0.0386 (19)	0.040 (2)	0.0016 (15)	0.0031 (16)	−0.0119 (16)
C39	0.034 (2)	0.055 (2)	0.053 (3)	0.0004 (18)	−0.0052 (19)	−0.008 (2)
C40	0.052 (3)	0.069 (3)	0.043 (2)	0.013 (2)	−0.005 (2)	−0.006 (2)
C41	0.055 (3)	0.066 (3)	0.045 (3)	0.011 (2)	0.006 (2)	0.010 (2)
C42	0.041 (2)	0.054 (2)	0.051 (3)	−0.0015 (19)	0.000 (2)	0.010 (2)

### Geometric parameters (Å, °)

**Table:**

Cd1—N4	2.328 (3)	C15—H15	0.9300
Cd1—O2^i^	2.336 (3)	C16—H16	0.9300
Cd1—N3	2.338 (3)	C17—C18	1.524 (6)
Cd1—O2	2.338 (3)	C18—C19	1.365 (6)
Cd1—O3	2.349 (3)	C18—C20	1.424 (6)
Cd1—O4	2.465 (3)	C19—C26	1.411 (6)
Cd1—O1	2.618 (3)	C19—H19	0.9300
Cd1—C17	2.717 (4)	C20—C21	1.416 (7)
O1—C1	1.229 (5)	C20—C25	1.433 (6)
O2—C1	1.274 (5)	C21—C22	1.381 (7)
O2—Cd1^i^	2.336 (3)	C21—H21	0.9300
O3—C17	1.252 (5)	C22—C23	1.394 (8)
O4—C17	1.255 (5)	C22—H22	0.9300
N1—C10	1.319 (5)	C23—C24	1.345 (8)
N1—C9	1.365 (5)	C23—H23	0.9300
N2—C26	1.309 (6)	C24—C25	1.407 (7)
N2—C25	1.366 (6)	C24—H24	0.9300
N3—C33	1.331 (5)	C26—C27	1.507 (6)
N3—C37	1.338 (5)	C27—C30	1.361 (7)
N4—C38	1.348 (5)	C27—C28	1.387 (7)
N4—C42	1.348 (5)	C28—C29	1.379 (8)
C1—C2	1.510 (5)	C28—H28	0.9300
C2—C3	1.367 (5)	C29—C32	1.383 (9)
C2—C4	1.420 (5)	C29—H29	0.9300
C3—C10	1.423 (5)	C30—C31	1.383 (8)
C3—H3	0.9300	C30—H30	0.9300
C4—C5	1.416 (6)	C31—C32	1.369 (9)
C4—C9	1.421 (5)	C31—H31	0.9300
C5—C6	1.365 (6)	C32—H32	0.9300
C5—H5	0.9300	C33—C34	1.382 (6)
C6—C7	1.398 (7)	C33—H33	0.9300
C6—H6	0.9300	C34—C35	1.363 (7)
C7—C8	1.352 (7)	C34—H34	0.9300
C7—H7	0.9300	C35—C36	1.380 (6)
C8—C9	1.417 (6)	C35—H35	0.9300
C8—H8	0.9300	C36—C37	1.395 (5)
C10—C11	1.500 (6)	C36—H36	0.9300
C11—C14	1.377 (6)	C37—C38	1.493 (5)
C11—C12	1.384 (7)	C38—C39	1.387 (6)
C12—C13	1.390 (7)	C39—C40	1.386 (6)
C12—H12	0.9300	C39—H39	0.9300
C13—C16	1.356 (8)	C40—C41	1.362 (7)
C13—H13	0.9300	C40—H40	0.9300
C14—C15	1.378 (7)	C41—C42	1.374 (6)
C14—H14	0.9300	C41—H41	0.9300
C15—C16	1.364 (9)	C42—H42	0.9300
			
N4—Cd1—O2^i^	88.20 (11)	C14—C15—H15	119.5
N4—Cd1—N3	70.50 (11)	C13—C16—C15	118.7 (5)
O2^i^—Cd1—N3	100.51 (10)	C13—C16—H16	120.6
N4—Cd1—O2	116.98 (10)	C15—C16—H16	120.6
O2^i^—Cd1—O2	74.31 (10)	O3—C17—O4	123.4 (4)
N3—Cd1—O2	170.24 (10)	O3—C17—C18	117.6 (4)
N4—Cd1—O3	139.30 (12)	O4—C17—C18	118.8 (4)
O2^i^—Cd1—O3	132.48 (11)	O3—C17—Cd1	59.7 (2)
N3—Cd1—O3	96.47 (11)	O4—C17—Cd1	65.0 (2)
O2—Cd1—O3	81.66 (10)	C18—C17—Cd1	163.5 (3)
N4—Cd1—O4	155.01 (11)	C19—C18—C20	118.0 (4)
O2^i^—Cd1—O4	81.67 (11)	C19—C18—C17	117.9 (4)
N3—Cd1—O4	88.89 (11)	C20—C18—C17	123.9 (4)
O2—Cd1—O4	82.22 (10)	C18—C19—C26	121.1 (4)
O3—Cd1—O4	54.54 (11)	C18—C19—H19	119.4
N4—Cd1—O1	81.20 (11)	C26—C19—H19	119.4
O2^i^—Cd1—O1	110.20 (10)	C21—C20—C18	125.4 (4)
N3—Cd1—O1	137.22 (11)	C21—C20—C25	117.5 (4)
O2—Cd1—O1	52.33 (9)	C18—C20—C25	117.0 (4)
O3—Cd1—O1	84.39 (11)	C22—C21—C20	121.0 (5)
O4—Cd1—O1	123.74 (10)	C22—C21—H21	119.5
N4—Cd1—C17	162.27 (12)	C20—C21—H21	119.5
O2^i^—Cd1—C17	106.23 (13)	C21—C22—C23	120.1 (5)
N3—Cd1—C17	96.26 (12)	C21—C22—H22	120.0
O2—Cd1—C17	77.65 (11)	C23—C22—H22	120.0
O3—Cd1—C17	27.41 (12)	C24—C23—C22	120.8 (5)
O4—Cd1—C17	27.49 (12)	C24—C23—H23	119.6
O1—Cd1—C17	102.66 (12)	C22—C23—H23	119.6
C1—O1—Cd1	86.3 (2)	C23—C24—C25	121.3 (5)
C1—O2—Cd1^i^	126.3 (2)	C23—C24—H24	119.3
C1—O2—Cd1	98.3 (2)	C25—C24—H24	119.3
Cd1^i^—O2—Cd1	105.69 (10)	N2—C25—C24	118.0 (4)
C17—O3—Cd1	92.9 (3)	N2—C25—C20	122.8 (4)
C17—O4—Cd1	87.5 (3)	C24—C25—C20	119.2 (5)
C10—N1—C9	118.3 (3)	N2—C26—C19	122.5 (4)
C26—N2—C25	118.3 (4)	N2—C26—C27	117.3 (4)
C33—N3—C37	119.2 (3)	C19—C26—C27	120.2 (4)
C33—N3—Cd1	122.8 (3)	C30—C27—C28	119.1 (5)
C37—N3—Cd1	118.0 (2)	C30—C27—C26	122.0 (5)
C38—N4—C42	118.8 (3)	C28—C27—C26	118.9 (5)
C38—N4—Cd1	118.2 (2)	C29—C28—C27	119.7 (6)
C42—N4—Cd1	122.9 (3)	C29—C28—H28	120.1
O1—C1—O2	123.1 (3)	C27—C28—H28	120.1
O1—C1—C2	120.5 (4)	C28—C29—C32	120.6 (6)
O2—C1—C2	116.4 (4)	C28—C29—H29	119.7
C3—C2—C4	119.1 (3)	C32—C29—H29	119.7
C3—C2—C1	119.3 (4)	C27—C30—C31	121.4 (6)
C4—C2—C1	121.5 (3)	C27—C30—H30	119.3
C2—C3—C10	120.3 (4)	C31—C30—H30	119.3
C2—C3—H3	119.9	C32—C31—C30	119.6 (6)
C10—C3—H3	119.9	C32—C31—H31	120.2
C5—C4—C2	124.6 (3)	C30—C31—H31	120.2
C5—C4—C9	118.8 (4)	C31—C32—C29	119.4 (6)
C2—C4—C9	116.6 (4)	C31—C32—H32	120.3
C6—C5—C4	120.7 (4)	C29—C32—H32	120.3
C6—C5—H5	119.6	N3—C33—C34	122.9 (4)
C4—C5—H5	119.6	N3—C33—H33	118.6
C5—C6—C7	120.2 (4)	C34—C33—H33	118.6
C5—C6—H6	119.9	C35—C34—C33	118.4 (4)
C7—C6—H6	119.9	C35—C34—H34	120.8
C8—C7—C6	120.8 (5)	C33—C34—H34	120.8
C8—C7—H7	119.6	C34—C35—C36	119.5 (4)
C6—C7—H7	119.6	C34—C35—H35	120.3
C7—C8—C9	121.1 (4)	C36—C35—H35	120.3
C7—C8—H8	119.5	C35—C36—C37	119.3 (4)
C9—C8—H8	119.5	C35—C36—H36	120.4
N1—C9—C8	118.2 (4)	C37—C36—H36	120.4
N1—C9—C4	123.5 (4)	N3—C37—C36	120.7 (4)
C8—C9—C4	118.3 (4)	N3—C37—C38	116.9 (3)
N1—C10—C3	122.1 (4)	C36—C37—C38	122.4 (4)
N1—C10—C11	116.6 (3)	N4—C38—C39	120.9 (4)
C3—C10—C11	121.3 (4)	N4—C38—C37	116.2 (3)
C14—C11—C12	117.9 (4)	C39—C38—C37	122.9 (3)
C14—C11—C10	119.8 (4)	C40—C39—C38	119.2 (4)
C12—C11—C10	122.3 (4)	C40—C39—H39	120.4
C11—C12—C13	120.2 (5)	C38—C39—H39	120.4
C11—C12—H12	119.9	C41—C40—C39	119.6 (4)
C13—C12—H12	119.9	C41—C40—H40	120.2
C16—C13—C12	121.2 (6)	C39—C40—H40	120.2
C16—C13—H13	119.4	C40—C41—C42	118.9 (4)
C12—C13—H13	119.4	C40—C41—H41	120.6
C11—C14—C15	120.9 (5)	C42—C41—H41	120.6
C11—C14—H14	119.6	N4—C42—C41	122.5 (4)
C15—C14—H14	119.6	N4—C42—H42	118.8
C16—C15—C14	121.1 (5)	C41—C42—H42	118.8
C16—C15—H15	119.5		

Symmetry codes: (i) −*x*+1, −*y*+1, −*z*.
